# Comparative ubiquitinome analysis reveals the roles of protein ubiquitination in the heat stress response from *Metarhizium robertsii*

**DOI:** 10.1128/aem.01468-25

**Published:** 2025-12-01

**Authors:** Jueping Song, Ali Raza, Hanyuan Chen, Guangshuo Li, Bo Huang, Zhangxun Wang

**Affiliations:** 1 Anhui Province Key Laboratory of Crop Integrated Pest Management, School of Plant Protection, Anhui Agricultural University 12486, Hefei, China; 2 National Collection of Plant-associated Microbes (Anhui), Anhui Agricultural University 12486, Hefei, China; 3 Anhui Province Key Laboratory of Microbial Pest Control, Anhui Agricultural University 12486, Hefei, China; Royal Botanic Gardens Kew, Surrey, United Kingdom

**Keywords:** *Metarhizium*, heat stress, ubiquitinome, protein ubiquitination, phosphoenolpyruvate carboxykinase

## Abstract

Protein ubiquitination plays a vital role in the stress response of diverse filamentous fungi. However, few reports are available on fungal insect pathogens, including *Metarhizium*. Here, we report a comparative ubiquitylome analysis of *Metarhizium robertsii* exposed to heat stress. The growth of *M. robertsii* was suppressed, and protein ubiquitination levels were markedly promoted during heat stress. Compared to the control treatment, there were 4,674 sites with differential ubiquitination, of which 3,419 lysine ubiquitination sites across 1,344 proteins were significantly upregulated, and 1,255 sites on 750 proteins were downregulated under heat stress. Further analysis showed that these proteins with upregulated modified sites were preferentially enriched in the phenylalanine, tyrosine, and tryptophan biosynthesis, pantothenate and CoA biosynthesis, and O-glycan biosynthesis pathways. Proteins with downregulated modified sites were significantly enriched in different pathways, including alanine, aspartate, and glutamate metabolism, pyruvate metabolism, and fatty acid biosynthesis. In particular, a key protein (phosphoenolpyruvate carboxykinase, MrPCK1, a central enzyme in gluconeogenesis and pyruvate metabolism) with five ubiquitination sites was identified, and functional analysis further revealed its regulatory role in heat stress tolerance of *M. robertsii*. Taken together, our findings suggest that *M. robertsii* may respond to heat stress not only through the canonical pathway of the proteasome but also by modulating specific metabolic pathways, including pyruvate metabolism (notably via MrPCK1) and potentially fatty acid biosynthesis. The results provide insights into the molecular mechanisms by which ubiquitination regulates the heat stress response in *M. robertsii* and contribute to our understanding of thermotolerance in filamentous fungi.

**IMPORTANCE:**

Entomopathogenic fungi such as *Metarhizium robertsii* are widely deployed as environmentally friendly biocontrol agents, yet their field performance is often limited by exposure to fluctuating and elevated temperatures. Although ubiquitination, a reversible post-translational modification that regulates protein stability, localization, and activity, is well known to orchestrate eukaryotic stress responses, its function in fungal heat adaptation has not been explored. To address this gap, we generated the proteome-wide ubiquitinome atlas of *M. robertsii* under thermal stress, cataloging modified sites across diverse metabolic and signaling pathways. Building on this global dataset, we demonstrate that ubiquitination of a key protein (phosphoenolpyruvate carboxykinase) involved in pyruvate homeostasis is essential for conidial thermotolerance in *M. robertsii*, thereby contributing to our understanding of the mechanism of heat stress adaptation in fungi. These findings provide a rich dataset that will inform future functional studies and guide the rational engineering or selection of more robust fungal strains for sustainable pest management.

## INTRODUCTION

Ubiquitination is a post-translational modification (PTM) that is involved in regulating the stability, activity, and localization of proteins within cells (1). It occurs through the covalent attachment of ubiquitin, a small protein, to lysine residues on the target proteins. This modification plays a central role in the control of protein degradation, functional regulation, and targeting of proteins to specific cellular compartments (2). The ubiquitin-proteasome system is required for maintaining cellular protein homeostasis, that is, the balance between synthesis, folding, and degradation of proteins, by controlling the degradation of misfolded, damaged, or unnecessary proteins, thereby ensuring proper protein turnover and functionality in cells (3).

Ubiquitination has a diverse array of biological functions, including the growth, development, and stress resistance of different fungi. For example, in *Saccharomyces cerevisiae*, ubiquitination plays an essential role in modulating mitochondrial homeostasis, specifically the quality control of mitochondrial proteins and the regulation of mitochondrial dynamics, ensuring proper energy production and organelle integrity (4), Golgi-endosome sorting to prevent toxin activity (5), methylation of histones for proper genome packaging (6), growth regulation via mitogen-activated protein kinase (MAPK) modulation (7), and in oxidative and heat stress responses (8, 9). In *Candida albicans*, ubiquitination regulates proteins involved in heat shock responses, including heat shock transcription factors (10). In *Magnaporthe oryzae*, ubiquitination plays essential roles in development, pathogenicity, stress response, and effector-mediated plant-pathogen interactions (11). Ubiquitination is also integral to fungal virulence in *Fusarium graminearum* (12, 13). In *Beauveria bassiana*, Ubr1-mediated ubiquitylation regulates asexual development, polar growth, and virulence-related cellular events (14). Beyond eukaryotes, ubiquitination-like systems have also been reported in prokaryotes (15). Bacteria employ small-protein modifiers such as Pup (prokaryotic ubiquitin-like protein) to tag proteins for proteasomal degradation, serving an analogous role in protein quality control under stress conditions. Such findings illustrate that ubiquitin-like regulation of protein stability is an evolutionarily conserved strategy across domains of life and highlight its broad relevance to stress adaptation (16). Although studies have highlighted the importance of ubiquitination in homeostasis, development, and response to stressors, the specific contributions of ubiquitination to heat stress in fungal insect pathogens remain poorly understood, including *Metarhizium*.

*Metarhizium robertsii*, an entomopathogenic fungus, is widely applied as a biocontrol agent in the management of insect pests. For *M. robertsii* to be effective in biocontrol applications, it must survive and function under varying environmental conditions, particularly fluctuating temperatures. Heat stress is widely reported to be one of the major impediments to the efficiency of pest control, except for virulence, in *Metarhizium* (17, 18). Heat shock proteins (HSPs), particularly HSP70 and HSP90 families, represent another critical component of the heat stress response across fungi, plants, and animals. In Mycobacterium, the bacterial proteasome activator Bpa mediates ATP-independent proteasomal degradation of the heat shock repressor under elevated temperatures, linking HSP regulation with proteasomal turnover (19). Recent evidence from *S. cerevisiae* and plants also implicates phosphoenolpyruvate carboxykinase and related enzymes in pyruvate metabolism as crucial regulators of metabolic reprogramming during heat stress. For instance, phosphoenolpyruvate carboxykinase contributes to gluconeogenesis and can compensate where pyruvate carboxylase activity is compromised in *S. cerevisiae* (20). Moreover, phosphoenolpyruvate carboxykinase is regulated post-translationally (e.g., by phosphorylation) and plays roles in balancing metabolic flux under stress in plants (21). Understanding how ubiquitination regulates such metabolic enzymes in *M. robertsii* is particularly relevant for sustainable pest management, where thermotolerance directly impacts the efficacy of fungal biocontrol agents. Specifically, our previous RNA-seq and functional analysis reports showed that several ubiquitin-associated genes (such as *MrUBI4* and *MrUbp4*) and pathways (including the proteasome pathway) are involved in the heat stress tolerance of *M. robertsii* (22–24). However, the response to heat stress is a complicated process involved in the regulation at diverse levels; therefore, it is required for the details of protein ubiquitination based on proteomic analysis of the heat stress response of *M. robertsii*.

Here, we aimed to investigate the role of ubiquitination in the heat stress response of *M. robertsii*, and the results identified ubiquitinated proteins in *M. robertsii* under heat stress conditions using proteomic profiling. Our findings highlight how ubiquitin-dependent modifications contribute to metabolic regulation and stress adaptation in *M. robertsii*, offering new insights into the molecular mechanisms underlying heat tolerance of this important fungal insect pathogen.

## RESULTS

### High temperature suppresses growth and increases the ubiquitination levels of total proteins in *M. robertsii*

**Fig 1:**
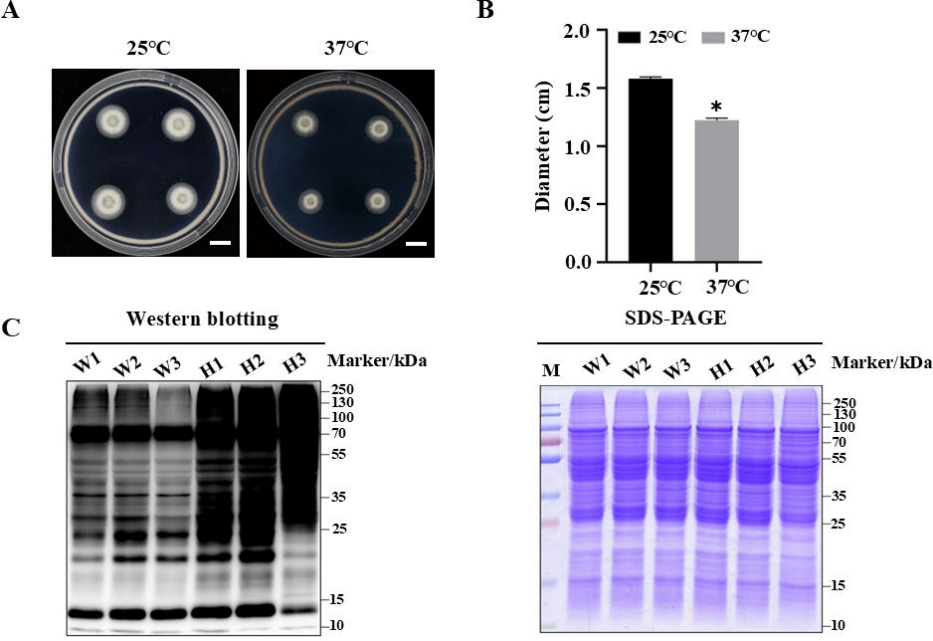
Evaluation of heat stress tolerance in *M. robertsii*. (**A**) Colony morphology of *M. robertsii* cultured on PDA at 25°C (control) and 37°C for 12 hours following pre-incubation at 25°C for 2.5 days. (**B**) Quantification of colony diameters (cm) under control (25°C) and heat stress (37°C) conditions. (**C**) Heat stress at 37°C for 180 min led to increased ubiquitination by western blotting analysis using anti-ubiquitin antibody (total proteins stained with Coomassie Brilliant Blue as control). “W” represents wild-type samples without high-temperature treatment, and “H” represents the wild-type sample subjected to high-temperature treatment at 37°C for 3 hours. Scale: 1 cm. Data are presented as means ± SD, the error bars represent the SD of three biological replicates. Tukey’s multiple comparison test was applied, and *P* < 0.05 was considered significantly different.

To investigate the response to thermo-stress in *M. robertsii*, we examined the growth of the wild-type strain under elevated temperature conditions. The *M. robertsii* colonies exposed to 37°C were significantly reduced compared to the control maintained at 25°C (control; Fig. 1A). The mean colony diameter under control conditions was 1.58 ± 0.02  cm, while heat-stressed colonies reached only 1.22 ± 0.02  cm, indicating a relative growth inhibition rate of 22.78% (Fig. 1B). This result suggests that high temperatures adversely affect *M. robertsii*. Subsequently, the results of western blotting revealed a pronounced increase in global ubiquitination levels of proteins after 180 min of exposure to 37°C (Fig. S1; Fig. 1C). Therefore, the cultures after 180 min of exposure to 37°C with three replicates were selected for the subsequent ubiquitylome analysis (Fig. 1C).

### Overview of the lysine ubiquitination in response to heat stress

**Fig 2:**
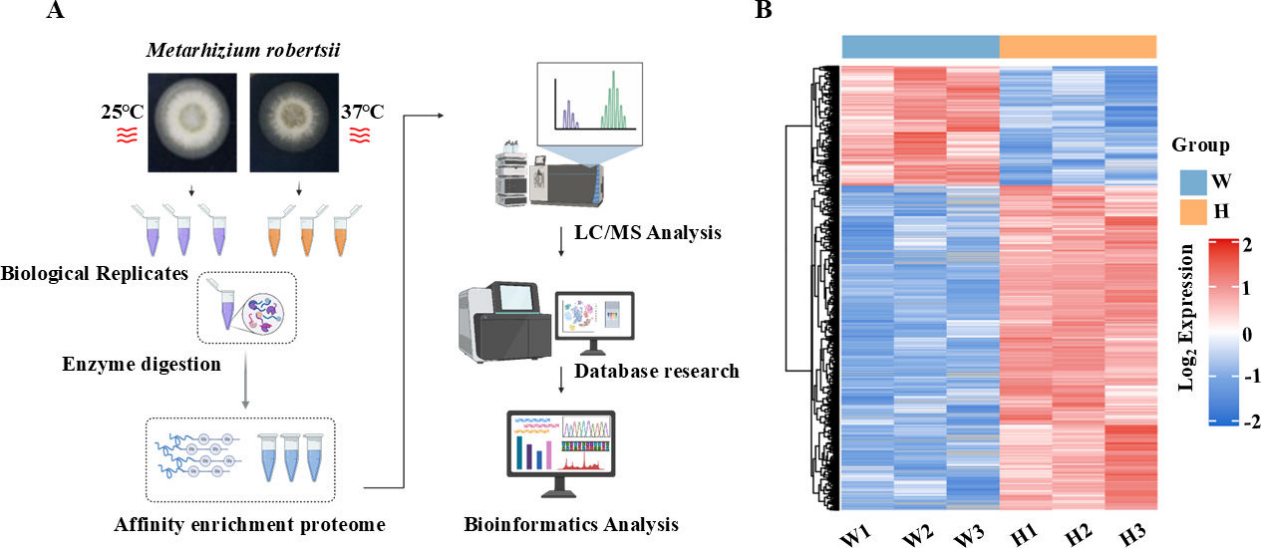
Proteome-wide identification and quantification of lysine ubiquitination in *M. robertsii* under heat stress. (**A**) Schematic representation of the experimental workflow: Lysine-ubiquitinated peptides were enriched using anti-K-ε-GG antibody-conjugated beads and analyzed via liquid chromatography-tandem mass spectrometry (LC-MS/MS) for comprehensive ubiquitinomics profiling. (**B**) Heatmap showing hierarchical clustering of significantly differentially ubiquitinated lysine sites under control (25°C) and heat stress (37°C) conditions. Each row represents a ubiquitination site, and color intensity reflects the relative abundance level (log_2_ fold change). Red indicates upregulation, and blue indicates downregulation. Data were normalized and clustered based on Euclidean distance. “W” represents wild-type samples without high-temperature treatment, and “H” represents the wild-type sample subjected to high-temperature treatment at 37°C for 3 hours.

To explore the global landscape of ubiquitination modifications in *M. robertsii* under heat stress, a label-free, quantitative ubiquitinome approach was applied. Lysine-ubiquitinated peptides were enriched using K-ε-GG antibody-conjugated beads and subjected to high-resolution mass spectrometry (Fig. 2A). This strategy led to the identification of 31,799 peptides, of which 22,727 were lysine modified (Fig. S2A; Table S1). The majority of the peptides ranged between 7 and 20 amino acids in length (Fig. S2B), which is a typical length distribution of tryptic peptides that is compatible with mass spectrometric detection. In total, 22,873 lysine ubiquitination (Kub) sites were mapped across 4,722 proteins, representing a substantial coverage of the ubiquitinated proteome in *M. robertsii*. Of these, 13,302 sites on 3,146 proteins were quantitatively assessed with high confidence (Fig. S2A; Table S1). Moreover, the sample quality was rigorously validated through comparative analysis of the intensity distributions of the modification sites, which showed tight alignment across replicates (Fig. S2C), indicating excellent reproducibility. For differential analysis, there are 4,674 sites that were differentially ubiquitinated by using the threshold of fold change >1.5, of which 3,419 lysine ubiquitination sites across 1,344 proteins were significantly upregulated, and 1,255 sites on 750 proteins were downregulated under 37°C treatment compared to the control (Fig. 2B; Fig. S2D; Table S2). Specifically, a heatmap of these differentially modified sites demonstrated that the number of upregulated modified sites was higher than that of downregulated ubiquitinated sites (Fig. 2B; Fig. S2D), suggesting that ubiquitination levels of total proteins were markedly augmented in response to heat stress.

### Motif analysis reveals sequence preferences around ubiquitinated lysine

**Fig 3:**
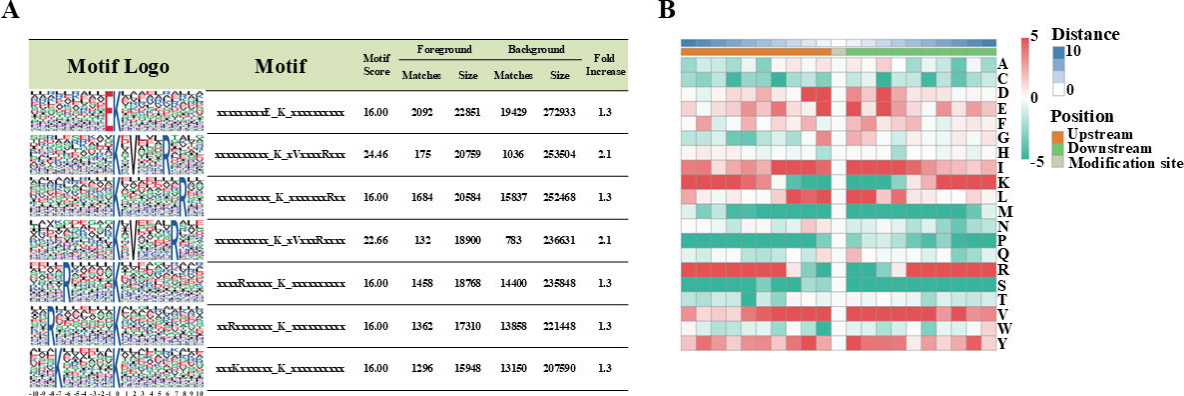
Sequence characteristics of lysine ubiquitination sites in *M. robertsii*. (**A**) Sequence motif analysis of peptides containing ubiquitinated lysine. Seven conserved motifs were identified using sequences ± 10 amino acids around the modified lysine residue (K). (**B**) Heatmap of amino acid frequency surrounding ubiquitination sites. The relative enrichment or depletion of amino acids within the ± 10-residue window is visualized, with stronger colors indicating higher frequency. Glutamate (E) and valine (V) were found to be significantly enriched, consistent with motif predictions.

To explore the sequence context of ubiquitination sites in *M. robertsii*, motif analysis was performed on peptides encompassing ±10 amino acids flanking each of the identified ubiquitinated lysine residues. Using the identified data set of high-confidence modification sites, seven motifs were extracted: EK, KxVxxxxR, KxxxxxxxR, KxVxxxR, RxxxxxK, RxxxxxxxK, and KxxxxxxK (where x indicates a random amino acid residue), where K represents the ubiquitinated lysine, and E, V, and R represent glutamate, valine, and arginine, respectively (Fig. 3A; Table S3). These motifs indicate a recurrent pattern of positively or negatively charged residues and hydrophobic residues surrounding the ubiquitination sites.

In addition to motif enrichment, the frequency profiles of amino acids surrounding ubiquitinated lysine were determined. The sequence logo generated from this analysis revealed a notable over-representation of E and V in positions adjacent to the modified lysine, further validating the observed motifs (Fig. 3B).

### Functional classification of differentially ubiquitinated proteins in the response to heat stress

To examine the potential roles of ubiquitination in response to heat stress in *M. robertsii*, Gene Ontology (GO) functional classification analysis was conducted based on these differentially ubiquitinated proteins. The results showed that under the biological process category, these ubiquitinated proteins were involved in cellular metabolic processes (11%), organic substance metabolic processes (11%), primary metabolic processes (10%), and nitrogen compound metabolic processes (10%; Fig. S3; Table S4). For the cellular component annotation, the majority of ubiquitinated proteins were localized to intracellular anatomical structures (21%), cytoplasm (18%), and organelles (17%; Fig. S3; Table S4). Regarding molecular functions, proteins exhibiting organic cyclic compound binding (15%), hydrolase activity (11%), and protein binding (10%) were predominant (Fig. S3; Table S4), suggesting that diverse biochemical activities were influenced by protein ubiquitination.

### KEGG pathway enrichment analysis for these differentially ubiquitinated proteins during heat stress

**Fig 4:**
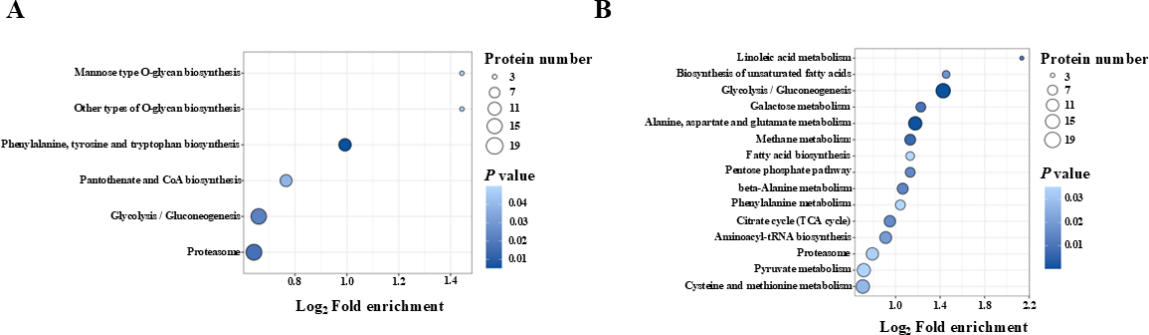
KEGG pathway enrichment analysis of differentially ubiquitinated proteins. (**A**) The significantly enriched pathways among ubiquitinated proteins with up-regulated modified sites. (**B**) The significantly enriched pathways among ubiquitinated proteins with down-regulated modified sites. Each dot represents one pathway; the size is proportional to the number of differentially ubiquitinated proteins mapped to that pathway, and the color reflects statistical significance.

**Fig 5:**
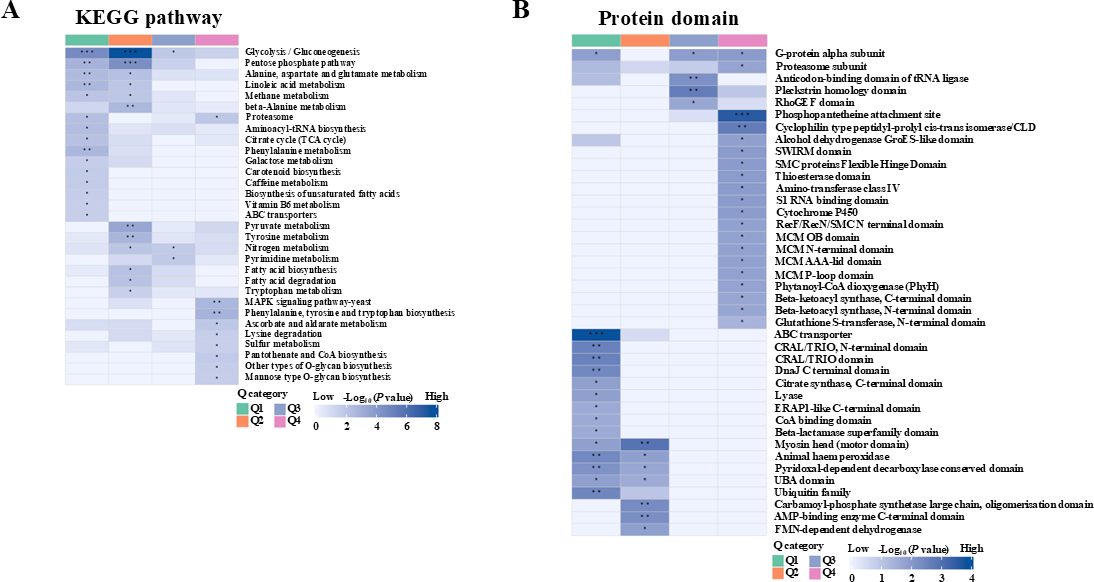
Clustering analysis of differentially ubiquitinated proteins. (**A**) KEGG pathway enrichment analysis of differentially ubiquitinated proteins based on cluster analysis. Pathways are arranged by similarity in enrichment profiles across heat stress, highlighting key biological processes and structural features associated with differential lysine ubiquitination in *M. robertsii*. (**B**) Protein domain enrichment analysis of differentially ubiquitinated proteins based on the cluster analysis. This highlights domain-level features of the ubiquitinated subset, reflecting their roles as conserved structural/functional units in stress-response and regulatory proteins. Colors reflect statistical significance, with darker colors indicating higher enrichment significance. The asterisks indicate statistical significance (* *P*  <  0.05, ** *P*  <  0.01, and *** *P*  <  0.001).

To further dissect the impact of heat-induced ubiquitination on these differentially ubiquitinated proteins in response to heat stress, Kyoto Encyclopedia of Genes and Genomes (KEGG) enrichment analysis was performed. These modified proteins with upregulated Kub sites were enriched in the pathways of phenylalanine, tyrosine, and tryptophan biosynthesis, pantothenate and CoA biosynthesis, and O-glycan biosynthesis (Fig. 4A), whereas proteins with downregulated Kub sites were enriched in other metabolic pathways, including alanine, aspartate, and glutamate metabolism, pyruvate metabolism, and fatty acid synthesis, including unsaturated fatty acid pathways (Fig. 4B). Noticeably, the important pathways, including glycolysis/gluconeogenesis and proteasome, were simultaneously regulated in both up- and downregulated Kub proteins (Fig. 4; Table S2), which was also supported by clustering analysis of these differentially modified proteins based on diverse levels of ubiquitination for the KEGG pathway (Fig. 5; Table S5). Moreover, other enrichment pathways (such as the MAPK signaling pathway) were also found to be enriched in upregulated Kub proteins according to the cluster analysis of these differentially ubiquitinated proteins (Fig. 5A; Table S5).

### Specific proteins with differentially ubiquitinated sites under heat stress

To determine which specific *M. robertsii* proteins were differentially ubiquitinated in response to heat stress, functional analysis for these proteins with differentially modified sites was performed. Our results showed that the proteins polyketide synthase (A0A0A1V0N2) and cysteine synthase (A0A0A1V1A3) were significantly upregulated under heat stress. Specifically, three modified sites (K1435, K1775, and K2229) for polyketide synthase were found in the top 10 upregulated Kub sites in these differentially ubiquitinated proteins. Conversely, several proteins, including the 26S proteasome regulatory complex (A0A014N2D3), E3 ubiquitin ligase (A0A014QWY1), and HSP90 protein (A0A0A1V9D8; Table S2), were identified as Kub sites that were significantly downregulated. Indeed, we also found that the corresponding protein domains, including the proteasome subunit, ubiquitin-associated domain, and ubiquitin family (Fig. 5B; Table S6), were significantly enriched according to further clustering analysis of the protein domain of these differentially ubiquitinated proteins. These proteins are associated with stress signaling, protein folding, and degradation, suggesting a role in cellular adaptation to heat stress.

### Functional validation for a ubiquitination-modified protein (MrPCK1) in response to thermo-stress

**Fig 6:**
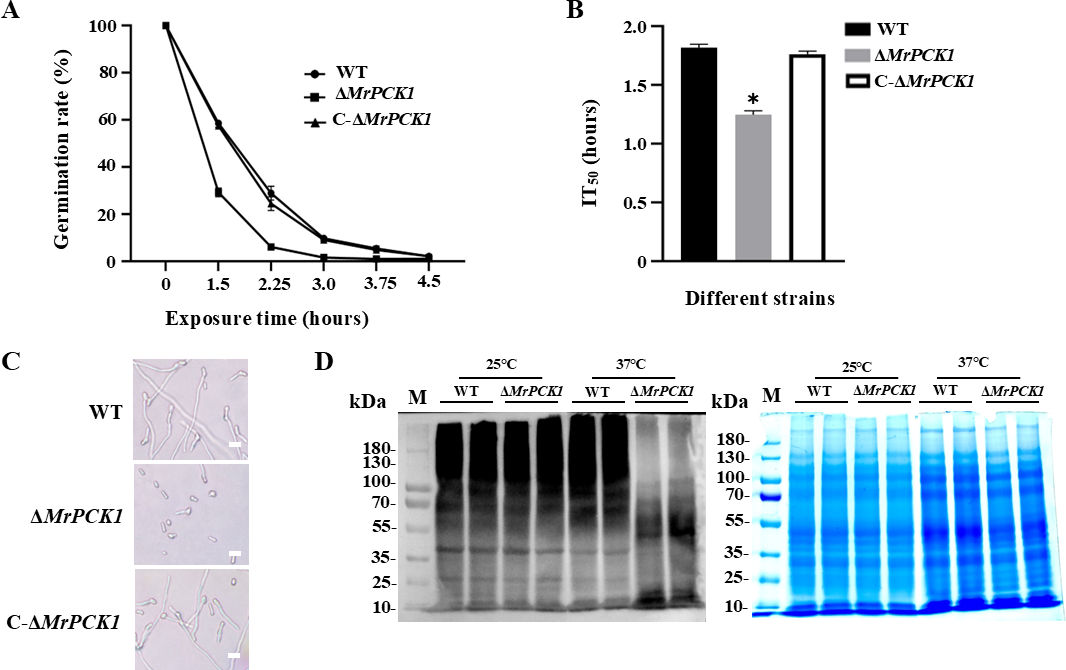
Effects of *MrPCK1* disruption on conidial tolerance to high temperature. (**A**) The conidial heat tolerance (conidial germination percentages) of respective strains was investigated during heat stress. Conidia were subjected to 45°C for varying durations, followed by incubation for 20 hours at 25°C to determine conidial germination. (**B**) The average 50% inhibition time (IT_50_) of respective strains during high temperature conditions. The error bars represent the SD. (**C**) Micrograph analysis for conidial germination of respective strains after cultured at 45°C for 2.25 hours, followed by a 20-hour incubation at 25°C. (**D**) Effects of *MrPCK1* disruption on ubiquitination levels of total proteins during heat stress. The ubiquitination levels of total proteins were detected by using an antiubiquitin antibody (left), with a Coomassie Brilliant Blue-stained gel shown as a control for total proteins (right). Scale: 10 µm. Data are presented as means ± SD, the error bars represent the SD of three biological replicates. Tukey’s multiple comparison test was applied, and *P* < 0.05 was considered significantly different.

Pyruvate plays an essential role in fungal resistance to heat stress (25, 26). In this study, a key enzyme involved in pyruvate metabolism, phosphoenolpyruvate carboxykinase (MrPCK1, A0A0A1V6R8), was identified with five differentially ubiquitinated sites following heat treatment (Table S7) and was thus selected for further functional analysis of thermo-stress. To investigate the effect of *MrPCK1* under high-temperature stress, a gene deletion was performed (Fig. S4), and one independent mutant and a complementation strain were selected to investigate the germination of conidia under heat stress. The results showed that the heat resistance of Δ*MrPCK1* conidia was significantly decreased, as evidenced by the lower conidial germination rates (exhibited as a shorter 50% inhibition time, IT_50_) than that of the control strain (Fig. 6A and B). Furthermore, microscopic analysis of the 2.25-hour heat-treated group confirmed that the germination rate of Δ*MrPCK1* conidia was indeed lower than that of the control strain (Fig. 6C). Moreover, compared with the control strains, the Δ*MrPCK1* mutant showed markedly reduced levels of ubiquitinated proteins after heat stress (37°C treatment), whereas there was no significant difference between the gene disruption and control strains under normal conditions (25°C incubation; Fig. 6D). Collectively, our results suggest that MrPCK1 is involved in the response to thermal stress in *M. robertsii*.

## DISCUSSION

Ubiquitination plays an essential role in cellular stress responses by modulating proteins that protect cellular integrity in response to environmental stressors such as heat, oxidative damage, and nutrient deprivation (27–29). In the present study, we examined the possible mechanisms by which ubiquitin-dependent modifications govern thermo-stress responses in *M. robertsii*. Our ubiquitinome analysis revealed a total of 31,799 peptides, with 22,727 lysine-modified peptides corresponding to 22,873 ubiquitination sites across 4,722 proteins, which provides a basis for further study of specific protein functions in *M. robertsii*. Furthermore, sequence preference analysis revealed seven motifs (EK, KxVxxxxR, and RxxxxxK), enriched in charged residues, including acidic (E), basic (R), and hydrophobic residues, such as valine (V), flanking ubiquitinated lysine in *M. robertsii*. Ubiquitination has been reported in diverse species, including wheat (30) and strawberry (31), where motifs such as EK, ExxxK, KD, and KE recur, indicating biochemical conservation of charged amino acids around ubiquitination sites. In mammals, analysis shows the preferential occurrence of hydrophobic residues (valine and leucine) adjacent to ubiquitinated sites (32). However, the longer motifs ending in R (e.g., KxxxxxxxR) appear to be unique to *M. robertsii*, suggesting species-specific diversification that potentially reflects adaptations to fungal proteome dynamics or thermal stress. These preferences suggest a potential sequence-dependent recognition mechanism by ubiquitin ligases in *M. robertsii* and may reflect the conserved structural or functional features required for lysine ubiquitination in response to heat stress. Our findings support a model where ubiquitination is guided by both conserved patterns and organismal tailoring of substrate recognition (33, 34).

There were 4,674 differentially ubiquitinated sites in 2,094 proteins during heat stress according to the criterion of fold change >1.5, and these ubiquitinated sites were found to be enriched in specific pathways, such as phenylalanine, tyrosine, and tryptophan biosynthesis, pantothenate and CoA biosynthesis, and O-glycan biosynthesis. The glycolysis/gluconeogenesis and proteasome pathways were found to be simultaneously involved in both up- and downregulated Kub proteins, which suggested two fine-tuned cellular processes for the response to heat stress. Indeed, the proteasome pathway has been identified as the classic pathway involved in the response to stress through the degradation of proteins of interest, whereas recent reports have shown that it is not involved in degrading damaged proteins during heat stress (35). Therefore, we speculated that it is also necessary to balance the canonical pathway for the heat stress response in *M. robertsii*. Phenylalanine, tyrosine, and tryptophan biosynthesis pathways play essential roles in thermotolerance and in the formation of a series of structural and defensive phenolic compounds in fungi (36, 37). Furthermore, pantothenate plays a key role in fungal responses to stress through its conversion to CoA (in *S. cerevisiae*) by supporting energy metabolism, maintaining membrane integrity, regulating heat shock protein synthesis, and modulating gene expression (38). In particular, O-glycan biosynthesis in fungi is a complex and tightly regulated process that contributes to various aspects of fungal biology. In *S. cerevisiae*, O-mannosylation, a type of O-glycosylation, is essential for proper protein folding and secretion (39). In *C. albicans*, the expression of genes involved in O-glycosylation is modulated during the transition from yeast-form to hyphal-form growth, reflecting the dynamic regulation of glycosylation pathways in response to environmental stimuli (40). Additionally, the MAPK signaling pathway was also enriched in upregulated protein sites upon heat stress, according to cluster analysis of the KEGG pathway. This is consistent with previous studies, which showed that heat stress responses usually include the activation of MAPK signaling (41–44).

Proteins with downregulated Kub sites were enriched in other important metabolic pathways, such as pyruvate metabolism and fatty acid synthesis, including unsaturated fatty acid pathways. Pyruvate metabolism is a central component of cellular energy production and metabolic flexibility. Heat stress may cause a shift in pyruvate usage, potentially favoring its conversion to acetyl-CoA, a key molecule that enters the trichloroacetic acid (TCA) cycle to support energy production and maintain cellular homeostasis (25). Previously, it was well established that heat stress also downregulated genes for pyruvate consumption in *M. robertsii*, and the results showed that the activities of pyruvate-metabolizing enzymes could be changed by heat treatment, resulting in pyruvate accumulation and thus increased tolerance to heat stress (26, 30, 45). In particular, our findings suggest that MrPCK1, a key enzyme in pyruvate synthesis, is involved in the response to thermal stress, which may be modulated by the ubiquitin-related pathway. Previous research has demonstrated the role of phosphoenolpyruvate carboxykinase in the heat stress response in animals, plants, bacteria, and fungi (46–49), which is consistent with that *MrPCK1* plays a role in regulating heat stress, likely through its involvement in gluconeogenesis or metabolic reprogramming (50). Heat stress in fungi activates MAPK cascades that can intersect with metabolic regulation. For example, in mammalian cells, stress-activated p38 MAPK phosphorylates ATF-2 and induces expression of phosphoenolpyruvate carboxykinase (PCK1) (51). Likewise, the Hog1 MAPK pathway is required for adapting to heat-induced stress in *M. robertsii* (45). These observations suggest that MrPCK1 may be regulated by MAPK-driven signaling branches (for instance, via ATF-2-like transcription factors or Hog1-mediated mechanisms) to align gluconeogenic flux with the heat stress response. In other words, stress-activated MAPKs could modulate MrPCK1 expression or stability as part of a coordinated network balancing energy production and stress protection. Similarly, heat shock factor 1 (HSF1) not only regulates chaperone expression but also integrates cellular metabolism; the loss of HSF1 depletes NAD^+^ and ATP and suppresses gluconeogenesis, while metabolic cues can activate HSF1 through phosphorylation (52). By analogy, MrPCK1 activity in *M. robertsii* may both influence and be influenced by the HSF network. Together, these potential interactions highlight MrPCK1 as a metabolic-signaling node in fungal heat stress adaptation.

Furthermore, proteins with downregulated ubiquitination sites were enriched in fatty acid biosynthesis pathways under heat stress, suggesting that lipid metabolism might be modulated as a part of the adaptive response. Previously, it was shown to synthesize smaller amounts of phospholipids with unsaturated fatty acid chains, directly affecting the plasma membrane composition under heat stress in *S. cerevisiae* (53). A similar trend was shown by *Torulopsis utilis,* increasing the biosynthesis of unsaturated fatty acids under heat stress (54). The ubiquitination modifications observed in enzymes related to fatty acid metabolism in *M. robertsii* may therefore indicate a potential regulatory connection between ubiquitin signaling and lipid remodeling. This adjustment likely serves to stabilize membrane fluidity and represents a resource-conserving strategy for coping with thermal stress (55, 56). However, this interpretation remains hypothetical, as our study did not directly focus on the amount or levels of enzymatic activity. Future studies integrating ubiquitinome data with lipidomic and biochemical analyses will be useful in understanding whether ubiquitination modulates fatty acid metabolism and membrane adaptation under heat stress.

In conclusion, our findings suggest that *M. robertsii* not only employs the canonical pathway of the ubiquitin-proteasome system to mitigate protein damage under heat stress, while also exhibiting ubiquitination modifications in metabolic pathways, such as pyruvate metabolism and fatty acid biosynthesis, that may contribute to thermotolerance. Our result provides a new insight into the ubiquitin-mediated regulation of heat stress response in *M. robertsii* and expands our understanding of stress response in filamentous fungi, which also provides a basis for improving the efficacy of entomopathogenic fungi in biocontrol applications. Further investigation should be conducted to biochemically validate specific ubiquitination sites and assess their functional effects. Additionally, future studies should also explore how ubiquitination interacts with other post-translational modifications, such as phosphorylation, acetylation, or SUMOylation. Such cross-talk may uncover additional regulatory layers that fine-tune protein stability, signaling, and metabolic adaptation under stress, thereby providing a more comprehensive understanding of fungal thermotolerance.

## MATERIALS AND METHODS

### Fungal strains, cultivation, and treatments

The wild-type (*M. robertsii* ARSEF 2575) strain, along with gene deletion and complementary strains, was cultured on potato dextrose agar (PDA) plates for 10 days. Conidia were then harvested and suspended in a 0.05% Tween 80 solution, followed by thorough shaking to ensure uniform dispersion. For heat stress tolerance analysis, 1 µL of conidial suspension (1 × 10⁷ conidia/mL) was spotted onto PDA plates and incubated at 25°C for 2.5 days (initial stage for conidial formation). Subsequently, plates were transferred to a 37°C incubator for 12 hours for heat stress, and heat tolerance was analyzed based on colony morphology and diameter. For sample collection of western and ubiquitinome analysis, aliquots (100 µL) of a conidial suspension (prepared from 10-day-old conidia at a concentration of 1 × 10⁶ conidia/mL) were inoculated onto PDA plates. After incubation at 25°C for 2.5 days, the control group was cultured at 25°C, whereas the heat treatment group was exposed to 37°C for 3 hours.

### Western blot analysis

Total protein for western blot analysis was prepared as previously described (24, 57), and 20 µg of total protein was separated by SDS-PAGE (8% gel, Yi Sheng, Shanghai, China). The proteins were then transferred to a nitrocellulose membrane (66485, Pall Life Sciences, MI, USA) for western blot analysis. The ubiquitination levels of total proteins were detected by using an anti-ubiquitin antibody (1:1,000 dilution, PTM-1106RM, PTM Bio, Hangzhou, China) and anti-rabbit secondary antibody (1:10000 dilution, 31460, Thermo Fisher Scientific, MA, USA).

### Protein preparation for ubiquitinome analysis and digestion

Ubiquitinome profiling was performed using three independent biological replicates (Fig. 2A). Protein extraction followed a modified protocol based on previous reports (58). Briefly, fungal samples (collected as described in the aforementioned section “Fungal strains, cultivation, and treatments”) were frozen in liquid nitrogen and ground into a fine powder. The homogenized material was suspended in phenol extraction buffer (4:1 ratio, buffer containing 10 mM dithiothreitol [DTT; Sigma-Aldrich], 1% protease inhibitor cocktail [Merck Millipore], and 50  µM PR-619 [Selleck Chemicals] to inhibit deubiquitinases). The mixture was disrupted by sonication and then combined with an equal volume of Tris-saturated phenol. After centrifugation at 5,500  ×  *g* for 10 minutes at 4°C, the phenolic phase was collected. Proteins were precipitated overnight by adding five volumes of methanol saturated with ammonium sulfate. The resulting pellet was harvested by centrifugation (10 minutes at 4°C) and sequentially washed with ice-cold methanol and acetone. The final protein pellet was solubilized in 8 M urea, and protein concentration was quantified using a BCA protein assay kit (Beyotime, Shanghai, China).

For digestion, equal amounts of protein were processed by adjusting the volume to match the lysate. TCA (Sigma-Aldrich) was added gradually to a final concentration of 20%, and the mixture was vortexed and incubated at 4°C for 2 hours to allow precipitation. The pellet was then collected by centrifugation at 4,500  ×  *g* for 5 minutes at 4°C and washed three times with chilled acetone. After drying, the pellet was reconstituted in 200 mM tetraethylammonium bromide (Sigma-Aldrich) and dispersed via sonication. Proteins were digested with trypsin at a 1:50 enzyme-to-protein ratio overnight. For peptide processing, DTT was added to a final concentration of 5 mM, and samples were incubated at 56°C for 30 minutes for reduction. Subsequently, alkylation was performed using 11 mM iodoacetamide (Sigma-Aldrich) in the dark at room temperature for 15 minutes.

### Enrichment of ubiquitin-modified peptides

For the isolation of lysine-ubiquitinated peptides, tryptic digests were dissolved in an immunoprecipitation buffer composed of 50  mM Tris-HCl (pH 8.0; Biosharp, Hefei, China), 100  mM NaCl (Sigma-Aldrich), 0.5% NP-40 (Sigma-Aldrich), and 1  mM EDTA (Sigma-Aldrich). The peptide mixture was then incubated overnight at 4°C with gentle rotation using anti-K-ε-GG antibody-conjugated agarose beads (PTM1104 and PTM Bio), which were pre-washed and equilibrated in the same buffer. After incubation, beads were washed thoroughly four times with the immunoprecipitation buffer to remove non-specific peptides, followed by two rinses with distilled water. Enriched peptides were eluted using 0.1% trifluoroacetic acid (Sigma-Aldrich), pooled, and lyophilized. The final eluates were desalted using C18 ZipTips (Millipore, Billerica, MA, USA) based on the manufacturer’s protocol before liquid chromatography-tandem mass spectrometry (LC-MS/MS) analysis.

### LC-MS/MS analysis

Tryptic peptides were reconstituted in Solvent A, consisting of 0.1% formic acid (Fluka) and 2% acetonitrile (Thermo Fisher Scientific), and directly loaded onto a reversed-phase analytical column (25 cm length, 100 µm inner diameter). Peptide separation was carried out using a nanoElute UHPLC system (Bruker Daltonics Inc., Billerica, MA, USA) with a binary solvent system: Solvent A (0.1% formic acid in 2% acetonitrile) and Solvent B (0.1% formic acid in 100% acetonitrile). The following gradient was applied: 6%–22% Solvent B from 0 to 18 minutes, 22%–30% from 18 to 22 minutes, 30%–80% from 22 to 26 minutes, and held at 80% from 26 to 30 minutes. The flow rate was maintained at a constant 450  nL/min throughout the run. The peptides were subjected to a capillary source followed by timsTOF Pro 2 mass spectrometry. The electrospray voltage applied was 1.65 kV, and data were collected using the data-independent acquisition (DIA)-parallel accumulation-serial fragmentation mode (59), with the secondary mass spectrometry scanning range set as 425–1,025 *m*/*z*.

### Database search, quantification, and bioinformatics analysis

DIA files were analyzed using Spectronaut software (version 18), with spectral matching conducted against the *M. robertsii* UniProt protein database (12,382 entries), which was combined with a reverse decoy database to estimate false discovery rates (FDRs). Proteolytic digestion was defined as Trypsin/P, allowing for up to four missed cleavages to account for potential incomplete digestion or contamination. Carbamidomethylation of cysteine residues was set as a fixed modification, while variable modifications included lysine ubiquitination, methionine oxidation, and N-terminal acetylation. Identification confidence was controlled using a 1% FDR threshold at the levels of protein, peptide, and peptide-spectrum match. For subsequent analysis, peptides with 7–52 amino acids were selected. For site-level analysis, a location probability of >0.75 was required for K-ε-GG site assignment. Only peptides and sites consistently detected in at least two biological replicates were retained. For the identification of differentially ubiquitinated sites, a fold change threshold of >1.5 and a *P*-value of <0.05 were applied, based on previously established criteria (60). For cluster analysis, ubiquitination sites were categorized into four quantitative groups based on fold change: Q1 (<0.5), Q2 (0.5–0.67), Q3 (1.5–2.0), and Q4 (>2.0).

To identify conserved Kub motifs, all sequences of Kub sites in *M. roberstii* proteins were analyzed using the Motif-X software (http://meme-suite.org/tools/momo). Domain annotation was conducted by using the InterProScan (http://www.ebi.ac.uk/interpro/). GO annotation was performed using EggNOG (http://eggnog5.embl.de/#/app/home). KEGG annotation was performed using the KEGG Automatic Annotation Server (KAAS; http://www.genome.jp/kegg/kaas/), and the significantly enriched KEGG pathways for proteins with differentially ubiquitinated sites were determined as *P* < 0.05, as described previously (61).

### Gene deletion

To generate a gene disruption construct for *MrPCK1* (MAA_02289/X797_000644), the 5′- and 3′-flanking regions (1,055 bp and 1,045 bp, respectively) were amplified from wild-type genomic DNA using 2 × Phanta Max Master Mix (Vazyme, Nanjing, China). The primers (GENERAL Biol, Chuzhou, China) used were MrPCK1-Up-F/MrPCK1-Up-R (with a *Pst* I restriction site) for the upstream region and MrPCK1-Dn-F/MrPCK1-Dn-R (with an *Xba* I site) for the downstream region, as listed in Table 1. The resulting PCR products were inserted into the pDHt-SK-bar plasmid-conferring glufosinate-ammonium resistance, using 2× MultiF Seamless Assembly Mix (ABclonal, Wuhan, China), yielding the knockout vector P-bar-*MrPCK1* (Fig. S4A). This vector was then used for fungal transformation to obtain the *MrPCK1* deletion strain (Δ*MrPCK1*), following previously established protocols (24, 57). For the construction of complementation strains (C-Δ*MrPCK1*), the full-length *MrPCK1* coding sequence along with its native promoter was amplified by using the primer pair C-*MrPCK1*-5F/C-*MrPCK1*-3R (with a *Pst* I site Table 1). The amplified fragment was cloned into the pDHt-ben vector, which confers resistance to benomyl. This complementation vector was introduced into the Δ*MrPCK1* background strain via fungal transformation (Fig. S4B). Successful integration of the constructs was confirmed by PCR using *MrPCK1*-L-F (P1)/*MrPCK1*-L-R (P2), while expression validation was performed by RT-PCR using *MrPCK1*-ID-F (P3)/*MrPCK1*-ID-R (P4) along with *GAPDH*-F/*GAPDH*-R as a control (Fig. S4C).

**TABLE 1:** Primers used in the study^*a*^

Gene	Primer name	Primer sequence (5'−3')	Purpose
*PCK1*	*MrPCK1*-Up-F	TTGATATCGAATTCCTGCAGAATGCCCACTGTGCTTTGTG, *Pst* I	For constructing knockout gene vector
	*MrPCK1*-Up-R	ACGGATCCCCCGGGCTGCAGGAAGCCAGAGCAACAGAGGA, *Pst* I	
	*MrPCK1*-Dn-F	TCTGATGAACTAGTTCTAGAACCCCATCGAGTACATCAAC, *Xba* I	
	*MrPCK1*-Dn-R	GCGGTGGCGGCCGCTCTAGACAAGTCGTTGGCCTTGTAAG, *Xba* I	
	*MrPCK1*-L-F (P1)	AACTCCTTCACACGCGACAC	For PCR detection
	*MrPCK1*-L-R (P2)	TGACAGGCAGGGAATCTTGG	
	*MrPCK1*-ID-F (P3)	ATCAACCGTGAGCGTGCTGT	For RT-PCR validation
	*MrPCK1*-ID-R (P4)	GATGCACTTGGCATAGCAAC	
	C-*MrPCK1*-5F	TTGATATCGAATTCCTGCAGGAACAGTCAATGCCCACTGT, *Pst* I	For constructing gene complementation vector
	C-*MrPCK1*-3R	GGCCCCCTGTCGAGCTGCAGACCAAGTCGAGGAAATTGAA, *Pst* I	
Bar	Bar-F	TCGTCAACCACTACATCGAGAC	For Bar plasmid validation
	Bar-R	GAAGTCCAGCTGCCAGAAAC	
Ben	Ben-F	GGTAACTCCACCGCCATCCA	For Ben plasmid validation
	Ben-R	GCAGGGTATTGCCTTTGGCACTT	
*GAPDH*	*GAPDH*-F	GACTGCCCGCATTGAGAAG	For RT-PCR validation (Inter control)
	*GAPDH*-R	AGATGGAGGAGTTGGTGTTG	

^*a*^ The enzyme cleavage sites used in the cloning procedure are underlined.

### Phenotypic assays

For phenotypic assays, experiments were conducted for different strains (WT, Δ*MrPCK1*, and C-Δ*MrPCK1*) according to our previous studies (24, 57). In brief, 1  mL of conidial suspension (1 × 10⁶ conidia/mL) was transferred into 1.5 mL microcentrifuge tubes and subjected to thermal stress at 45°C for a predetermined duration (0, 1.5, 2.25, 3, 3.75, and 4.5 hours). After heat exposure, a 10  µL aliquot of each suspension was spotted onto PDA plates. The plates were cultured at 25°C for 20  hours, after which germinated and ungerminated conidia were counted under a microscope. The time required to achieve 50% inhibition of germination (IT₅₀) was determined to assess heat sensitivity. Additionally, conidial germination morphology at specific post-treatment intervals was examined microscopically, as described in previous reports (62).

### Statistical analysis

Data for phenotypic analysis were expressed as mean ± SD of at least three biological replicates, as described previously (62). Statistical analyses were performed using IBM SPSS Statistics software, version 24.0. One-way analysis of variance (ANOVA) was applied to evaluate differences among treatments. Tukey’s multiple comparison test was used, and *P* value < 0.05 was considered significantly different.

## Data Availability

Mass spectrometry-based proteomic data were deposited in ProteomeXchange under accession number PXD065615). The *Metarhizium robertsii* strains, including the wild type, mutant, and complemented strains, are available upon request (luckywang2002@163.com).

## SUPPLEMENTAL MATERIAL

Supplemental figures aem.01468-25-s0001.pdf Figures S1 to S4.

Supplemental tables aem.01468-25-s0002.xlsx Tables S1 to S7.

ASM does not own the copyrights to Supplemental Material that may be linked to, or accessed through, an article. The authors have granted ASM a non-exclusive, world-wide license to publish the Supplemental Material files. Please contact the corresponding author directly for reuse.
