# Supplementary material for: Comparative ubiquitinome analysis reveals the roles of protein ubiquitination in the heat stress response from Metarhizium robertsii
Source: Appl Environ Microbiol. 2025 Dec 1;91(12):e01468-25. doi: 10.1128/aem.01468-25 (PMC12724308; doi:10.1128/aem.01468-25)
Supplement: Supplemental figures — Figures S1 to S4. [file aem.01468-25-s0001.pdf]

SUPPLEMENTARY MATERIAL FOR

Comparative ubiquitinome analysis reveals the roles of protein ubiquitination in the heat stress response from *Metarhizium robertsii*

Jueping Song[1, 2, 4], Ali Raza[1, 2, 4], Hanyuan Chen [1, 2], Guangshuo Li[1, 2], Bo Huang[2,3] and Zhangxun Wang [1, 2, *]

[1] Anhui Province Key Laboratory of Crop Integrated Pest Management, School of Plant Protection, Anhui Agricultural University, Hefei, 230036, China.

[2] National Collection of Plant-associated Microbes (Anhui), Anhui Agricultural University, Hefei, 230036, China.

[3] Anhui Province Key Laboratory of Microbial Pest Control, Anhui Agricultural University, Hefei, 230036, China.

[4] These authors have contributed equally to this study.

[*] Corresponding author.

Zhangxun Wang

Email: luckywang2002@163.com

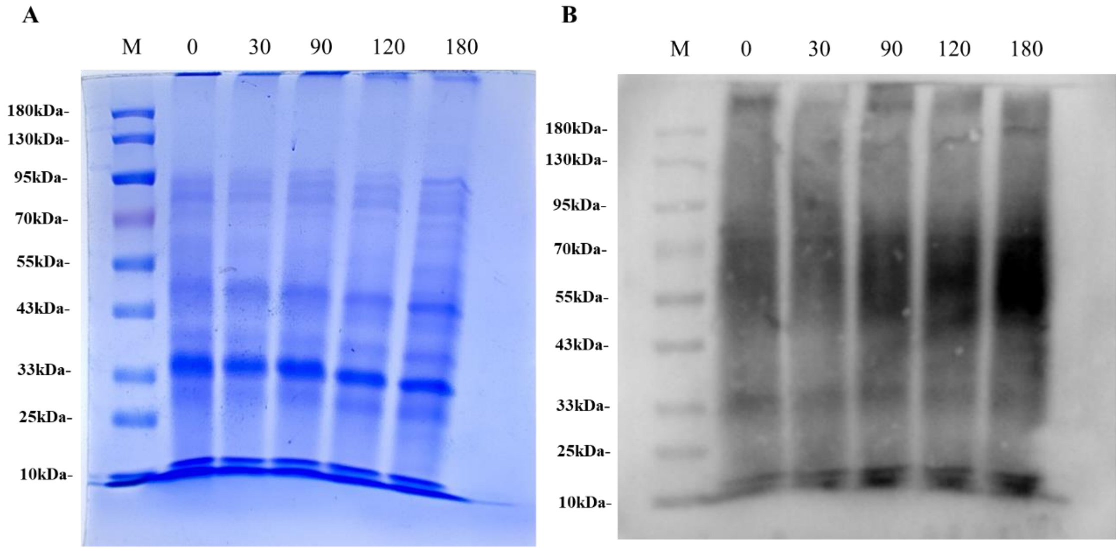

FIG S1 The impact of heat treatment on the ubiquitination levels.

(A) Total proteins of different treatment times treated by high temperature conditions were separated by SDS-PAGE gel, and stained with Coomassie brilliant blue (as control for total proteins).

(B) Western blotting analysis of total protein ubiquitination using anti-ubiquitin antibody. Time gradient heat treatment of hyphae at 37°C revealed a gradual increase in total ubiquitination levels with prolonged exposure duration, as detected by Western blotting.

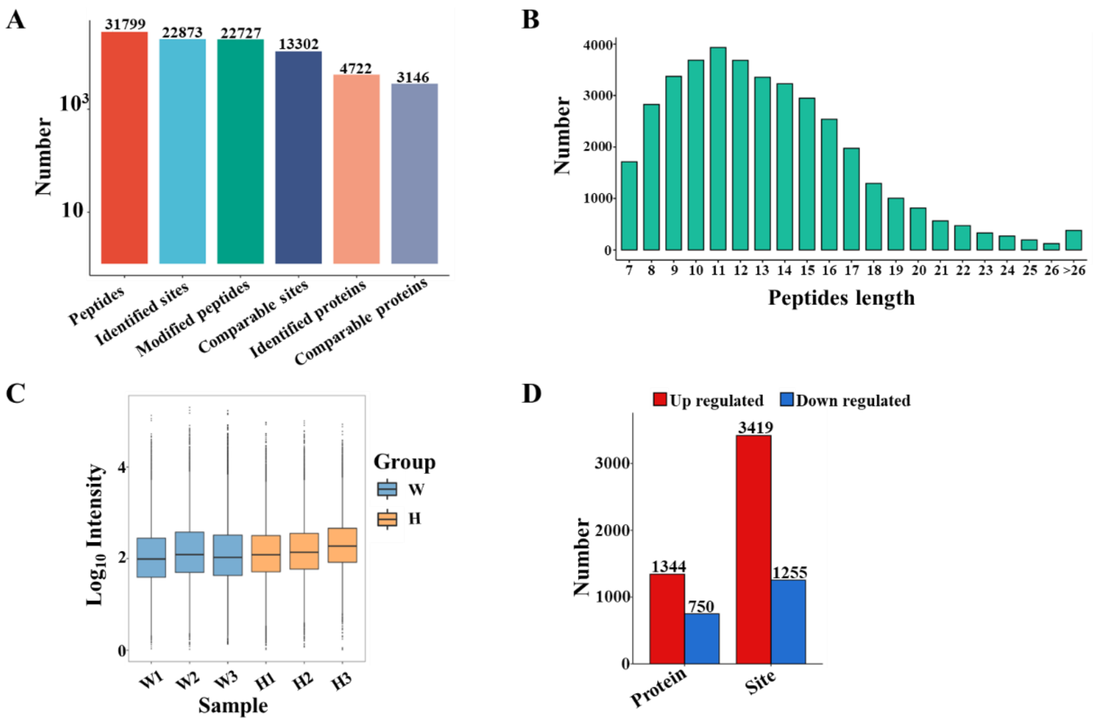

FIG S2 Overview of protein ubiquitination modification analysis.

(A) The number of peptides, modification sites, and proteins identified in ubiquitination modification omics analysis. The false positive rate (FDR) for protein, peptide, and PSM identification is 1%.

(B) Distribution of peptide length. The identified peptides ranged from 7 to 20 amino acids in length, consistent with the typical fragmentation patterns of enzymatic digestion and mass spectrometry. This peptide length distribution meets quality control criteria for mass spectrometric identification.

(C) Analysis of intensity values of modification sites. The sample mean is on the same horizontal line, indicating good sample quality. "W" represents wild-type samples without high-temperature treatment, and "H" represents the wild-type sample subjected to high temperature treatment at 37°C for 3 hours.

(D) Statistical analysis of the number of ubiquitination differential modification sites. When the *p* value is less than 0.05, a change in differential expression level exceeding

1.5 is considered a significant upregulation threshold, and a change level less than 1/1.5 is considered a significant downregulation threshold.

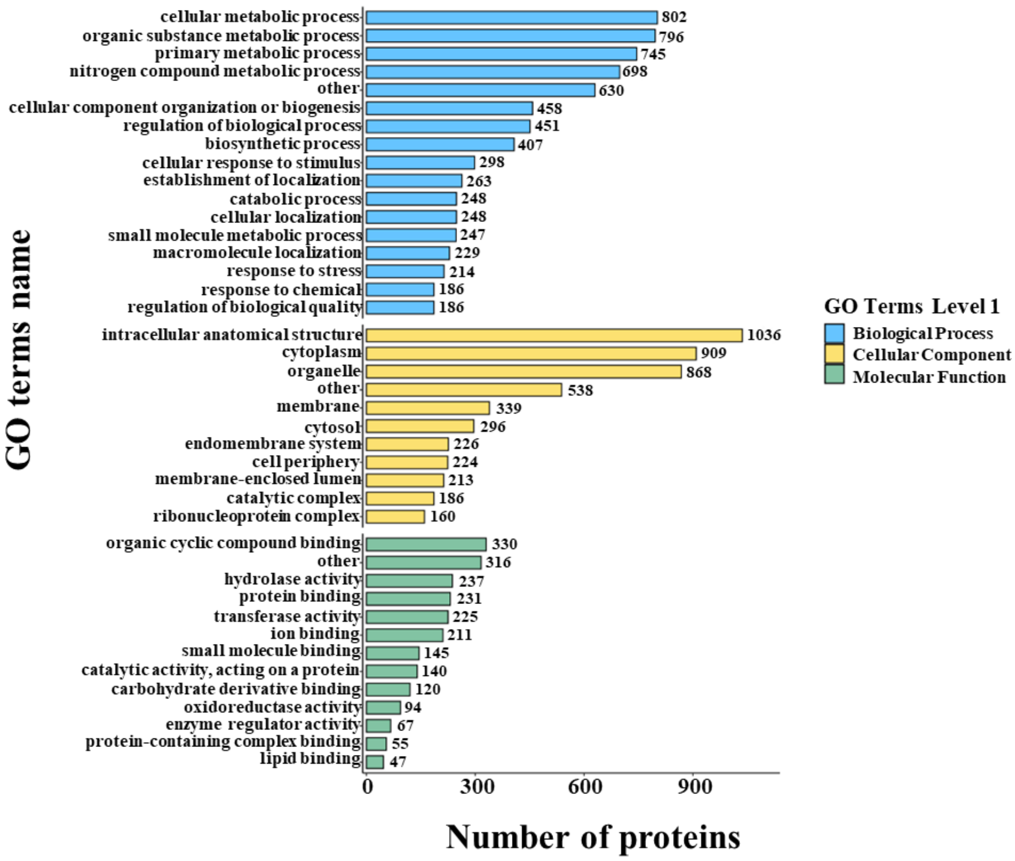

FIG S3 Functional classification of differentially modified proteins.

GO-based analysis of all quantified proteins. The *X* axis represents the number of differentially modified proteins in the classification, and the *Y* axis represents the secondary functional classification in the primary classification of GO. Different colors represent the first level classification of GO.

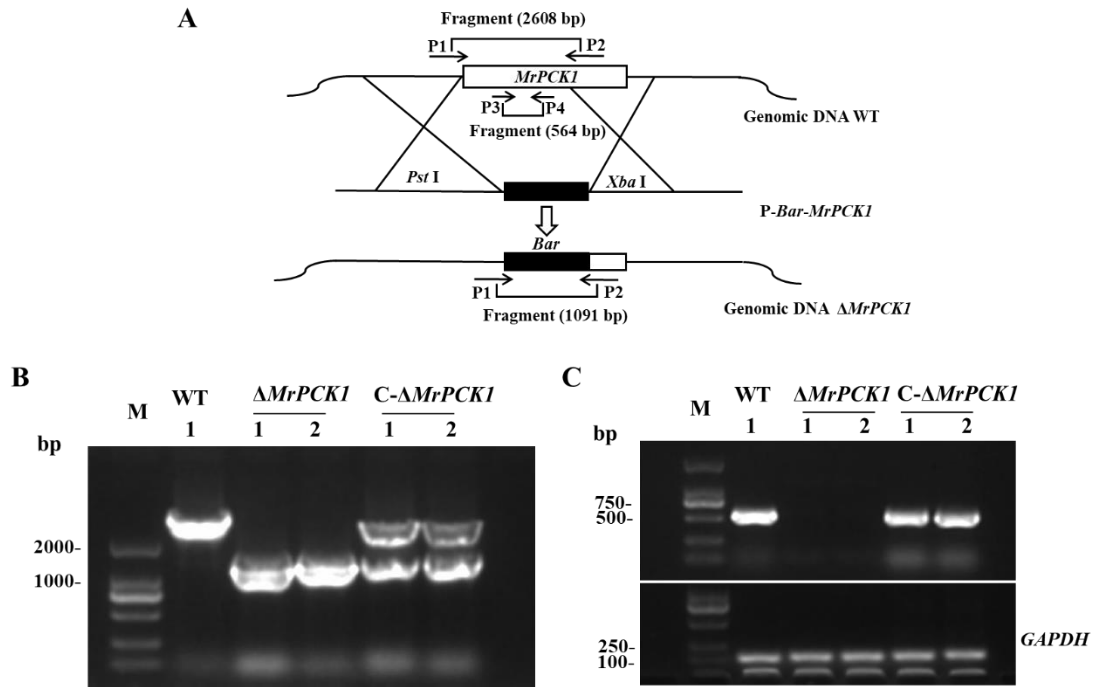

FIG S4 Gene disruption and complementation of *MrPCK1*.

(A) Schematic diagram of *MrPCK1* gene disruption via homologous recombination. Within this diagram, the 5' and 3' segments of *MrPCK1*, (1015 and 1005 bp) along with the overlapping regions in the P-Bar-MrPCK1 (recombinant vector), are employed for the precise replacement of a portion of *MrPCK1* with a 948-base pair Bar plasmid. The directional arrows demarcate the corresponding regions with clarity.

(B) PCR verification for Δ*MrPCK1* was conducted using primers P1/P2 (Table 1). Bands of 2608 base pairs were successfully amplified from the WT, while bands of 1091 bp were successfully amplified from the Δ*MrPCK1*, bands of 2608 and 1091 bp were successfully amplified from the C-Δ*MrPCK1* (Because the construction of the Ben plasmid containing the *MrPCK1* was transformed into the Δ*MrPCK1*, which includes both the Bar plasmid sequence and the *MrPCK1* sequence).

(C) RT-PCR analysis for *MrPCK1* expression. Using cDNA as a template and primers P3/P4 (Table 1) for verification, bands of 564 bp were successfully amplified from the WT and C-Δ*MrPCK1*, while no amplification was observed in Δ*MrPCK1*. Additionally, the expression level of glyceraldehyde-3-phosphate dehydrogenase (*GAPDH*, MAA_07675, Table 1) was employed as an internal control.
